# Sleep duration and breast cancer incidence: results from the Million Women Study and meta-analysis of published prospective studies

**DOI:** 10.1093/sleep/zsaa166

**Published:** 2020-09-04

**Authors:** Angel T Y Wong, Alicia K Heath, Tammy Y N Tong, Gillian K Reeves, Sarah Floud, Valerie Beral, Ruth C Travis

**Affiliations:** 1 Cancer Epidemiology Unit, Nuffield Department of Population Health, University of Oxford, Oxford, UK; 2 School of Public Health, Faculty of Medicine, Imperial College London, London, UK

**Keywords:** sleep duration, breast cancer, prospective, meta-analysis

## Abstract

**Study Objectives:**

To investigate the association between sleep duration and breast cancer incidence, we examined the association in a large UK prospective study and conducted a meta-analysis of prospective studies.

**Methods:**

In the Million Women Study, usual sleep duration over a 24-h period was collected in 2001 for 713,150 participants without prior cancer, heart problems, stroke, or diabetes (mean age = 60 years). Follow-up for breast cancer was by record linkage to national cancer registry data for 14.3 years on average from the 3-year resurvey. Cox regression models yielded multivariable-adjusted breast cancer relative risks (RR) and 95% confidence intervals (CIs) for sleep duration categories. Published prospective studies of sleep duration and breast cancer risk were included in a meta-analysis, which estimated the inverse-variance weighted average of study-specific log RRs for short and for long versus average duration sleep.

**Results:**

After excluding the first 5 years to minimize reverse causation bias in the Million Women Study, 24,476 women developed breast cancer. Compared with 7–8 h of sleep, the RRs for <6, 6, 9, and >9 h of sleep were 1.01 (95% CI, 0.95–1.07), 0.99 (0.96–1.03), 1.01 (0.96–1.06), and 1.03 (0.95–1.12), respectively. In a meta-analysis of 14 prospective studies plus the Million Women Study, including 65,410 breast cancer cases, neither short (RR < 7 h = 0.99 [0.98–1.01]) nor long (RR > 8 h = 1.01 [0.98–1.04]) versus average duration sleep was associated with breast cancer risk.

**Conclusions:**

The totality of the prospective evidence does not support an association between sleep duration and breast cancer risk.

**Statement of Significance**

We examined sleep duration and breast cancer risk in a large UK cohort with a mean follow-up of 14.3 years and 24,476 incident cases. To our knowledge, this is the largest individual prospective analysis published to date. We excluded the first 5 years of follow-up in our analyses to minimize potential reverse causation bias, whereby pre-clinical disease might cause an increase or decrease in sleep duration. The findings suggested no association between sleep duration and breast cancer risk. The findings were robust across sensitivity analyses. We performed a meta-analysis of 15 prospective studies including our study, with a total of 65,410 breast cancer cases. The totality of the prospective evidence does not show that breast cancer risk varies by sleep duration.

## Introduction

Short sleep duration has been hypothesized to increase breast cancer risk [1–15]. The light-at-night hypothesis suggests that exposure to artificial light at night may affect the circadian system and suppress melatonin production, which may lead to increased estrogen production and a higher risk of breast cancer [15]. In this way, shorter habitual sleep duration can be considered as a surrogate for greater long-term exposure to artificial light at night in most societies. However, it remains unclear if overnight or total sleep duration is associated with breast cancer risk.

Results from meta-analyses on the association of sleep duration with breast cancer risk are inconsistent [16, 17]. While a 2017 meta-analysis reported a non-linear association between sleep duration and breast cancer risk, a 2018 categorical meta-analysis of sleep duration and cancer risk did not find an association of breast cancer risk for short and for long sleep, versus average sleep duration, in a subgroup analysis [16, 17]. However, both meta-analyses did not differentiate between results from studies with prospective and retrospective study designs. In retrospective studies, women with breast cancer know their diagnosis and controls know that they do not have the disease, and this might differentially affect their reporting of past activities. By contrast, in prospective studies information about sleep and other factors is collected before cancer is diagnosed and hence is less susceptible to reporting bias.

We aimed to examine the association between usual sleep duration in a 24-h period and breast cancer risk using data from the large-scale prospective UK Million Women Study. We excluded the first 5 years of follow-up to minimize possible reverse causation bias, whereby preclinical cancer might cause changes in sleeping patterns. To put published results into context, we performed a systematic review and meta-analysis of prospective data on the association of 24-h or overnight sleep duration with breast cancer risk.

## Methods

### Million Women Study

The details of the Million Women Study have been previously described [18]. Briefly, 1.3 million women aged 50–64 years were recruited through the UK Breast Cancer Screening Programme during 1996–2001. Participants were resurveyed every 3–5 years after the recruitment surveys. Follow-up for incident cancer was achieved via record linkage to cancer registries. Ethical approval was granted by the Anglia and Oxford Multi-Centre Research Ethics Committee. All participants gave written consent. Data access policies for the Million Women Study are available via the study website (http://www.millionwomenstudy.org/).

### Collection of self-reported sleep duration and covariates

The current analyses were based on the 3-year resurvey questionnaire completed in 1999–2005, which included questions on sleep patterns for the first time [19]. Women were asked “about how many hours sleep do you get in every 24 hours? (please include naps)” and hours of sleep were coded in integers. We defined the valid range of 24-h sleep duration as 1–23 h. There is no consensus on categorization of sleep duration or the definition of “normal” hours of sleep [16]. As similar numbers of women reported 7 and 8 h of sleep, women were categorized as reporting <6, 6, 7–8 (reference), 9, and >9 h of sleep. Reproducibility of 24-h sleep duration was assessed in a subset of women who completed the baseline questionnaire twice on average 1.7 years apart using Cohen’s kappa statistics and a Bland–Altman plot.

Nearly all women were postmenopausal at baseline. Other personal characteristics used in these analyses were collected from the 3-year resurvey questionnaires, except for region of residence, educational attainment, Townsend deprivation index, strenuous exercise, age at menarche, height, age at first birth, and parity, which were recorded at the time of recruitment. Responses of “not known,” “not sure,” and “not answered” were treated as missing values. Information was missing for less than 10% for each covariate, apart from alcohol intake (12%).

#### Endpoint, exclusions, censoring

Information on cancer registrations and deaths, including cause-specific details, coded to the 10th revision of the International Classification of Diseases (ICD-10) [20], were obtained by record linkage to National Health Service (NHS) databases from NHS Digital in England and Information Services Division Scotland in Scotland. The main outcome for these analyses was first registration of invasive breast cancer (ICD-10 C50) or death attributed to breast cancer (ICD-10 C50).

Among participants who returned the 3-year questionnaire and did not have a prior cancer or breast carcinoma in situ registration (except non-melanoma skin cancer), we further excluded women: (1) with missing data on sleep patterns or an invalid sleep duration; (2) reporting use of sleeping pills or with a prior hospital diagnosis of insomnia, hypersomnia, or sleep apnea (as their reported sleep duration was not likely to reflect their habitual sleep duration); or (3) with a previous history of stroke, diabetes, or heart problem. This left the final sample of 713,150 women for the analysis (Supplementary Figure S1).

### Statistical analysis

Cox regression models using time in study as the underlying time variable estimated hazard ratios, henceforth called relative risks (RRs) and 95% confidence intervals (CIs) of sleep duration categories. Person-years were calculated from the date the 3-year baseline questionnaire was completed to the date of first incident invasive cancer (except non-melanoma skin cancer [ICD-10 C44]) date of death, date of loss to follow-up, or the end of follow-up (December 31, 2016 in Scotland and December 31, 2017 in England), whichever was earliest. If women had a diagnosis of any other cancer type or breast carcinoma in situ (ICD-10 D05), they were censored at the date of that cancer registration. To minimize reverse causation bias, we excluded the first 5 years of follow-up.

Based on previous analyses of breast cancer in the Million Women Study [21, 22], the Cox regression model was stratified by year of completion of the 3-year questionnaire and year of birth, and adjusted for region of residence (10 regions), Townsend deprivation index (fifths) [23], educational attainment (tertiary qualifications, secondary qualifications, technical qualifications, no qualifications and left after school leaving age, no qualifications and left before school leaving age), body mass index (BMI) (<22.5, 22.5–24.9, 25.0–27.4, 27.5–29.9, 30.0–32.4, 32.5–34.9, ≥35.0 kg/m^2^), alcohol intake (none or <1, 1–3, 4–6, ≥7 drinks per week), ever use of hormone replacement therapy (HRT) (never, past, current for <10 years, current for 10+ years), strenuous exercise per week (never/rarely, less than once per week, at least once per week), smoking status and number of cigarettes per day (never, past, current: 1–14 cigarettes/day, current: ≥15 cigarettes/day), age at menarche (<12, 12–13, ≥14 years), parity and age at first birth (nulliparous, 1–2/<25, 1–2/≥25, ≥3/<25, ≥3/≥25 years), family history of breast cancer (yes, no), and height (<160, 160–165.9, ≥166 cm). As nighttime sleep duration (rather than 24-h total sleep duration) may be more relevant to the risk of breast cancer in terms of the biological hypothesis, and daytime napping was found to be associated with breast cancer in a previous analysis in the Million Women Study [21], we adjusted for frequency of daytime napping (never/rarely, sometimes, and usually) as a potential confounder. Women with missing or unknown values for an adjustment variable were grouped into a separate category for that variable.

In the multivariable-adjusted model, we tested for trend by entering sleep duration as a continuous variable, with the value in each group being replaced by mean sleep duration in repeat questionnaire completed an average 1.7 years after baseline in order to correct for regression dilution bias (i.e. 5.6, 6.4, 7.4, 8.4, and 9.0 h, respectively, for categories <6, 6, 7–8, 9, >9 hours of sleep, which show the expected regression to the mean with repeat measurements).

Sensitivity analyses were done to assess the association only in women who reported rarely/never daytime napping, to assess the effects of reverse causation by restricting analyses to women who reported good or excellent self-rated health, and to assess the effects of missing data by conducting a complete case analysis. As there is no widely accepted valid range of sleep duration, we performed a sensitivity analysis with a more restricted range of valid sleep duration of 4–20 h, which further excluded 819 women (primarily women who reported very short sleep duration). The Schoenfeld residual test did not suggest violation of the proportional hazards assumption in the multivariable-adjusted model.

### Search strategy for systematic review

Supplementary Table S1 shows the search terms intended to search for prospective studies that examined sleep duration (including napping) with breast cancer risk in Embase and MEDLINE from inception to February 14, 2019.

#### Study selection

Titles and abstracts of records identified from the search strategy were screened by two independent reviewers (ATYW, AKH/RCT). An article was included if (1) it reported on an original research study in humans, (2) sleep duration or daytime napping was the exposure, (3) incident breast cancer was the outcome, (4) the sample was recruited entirely from the general population, and (5) the study had a prospective study design. All experimental, case–control, or cross-sectional studies, letters, or studies with insufficient data on the number of cases, multivariable-adjusted RR, and 95% CI were excluded. Similarly, full texts of the potentially relevant articles were screened independently using the same criteria. For multiple reports from the same cohort, the complete report with the longest follow-up period was included. Disagreements were resolved by discussion. The reference lists of included studies were searched to identify any additional relevant publications. Subsequent to the completion of this literature search, two more analyses of sleep duration with breast cancer risk were published from the Multiethnic cohort and UK Biobank [14, 24]. No breast cancer RR estimates across categories of sleep duration were available in the analyses of UK Biobank [24]. Hence, we only incorporated the results from the Multiethnic cohort into our meta-analysis.

#### Data extraction

Odds ratios and hazard ratios were regarded as RRs based on the rare disease assumption. Study characteristics were extracted from the included articles, whenever available: the study cohort, region of the study, year of recruitment, year of baseline when sleep duration was measured, follow-up period, analyzed sample size, mean age, exclusion criteria, outcome ascertainment, exposure assessment and categories, covariates adjusted for, number of cases, and multivariable-adjusted RRs and 95% CIs of non-reference categories of sleep duration with breast cancer risk, and those stratified by menopausal status. Multivariable-adjusted estimates with the exclusion of early follow-up periods or cases diagnosed soon after baseline were also extracted. Only estimates of weekday sleep duration were combined in the meta-analysis, as weekday sleep duration more likely reflects usual or habitual sleep duration. Study characteristics were presented according to nighttime sleep duration or total sleep duration. Each study was scored for risk of bias according to seven items covering three areas: selection of participants and exposure, comparability of the cohorts, and outcome ascertainment [25]. Representativeness of the exposed cohort was not assessed, as the interval validity of the association is likely to be generalizable even when the cohort is not a representative sample of the general population, provided that the range of exposure is sufficiently wide.

#### Statistical analysis

Our meta-analysis compared breast cancer risk for short (<7 h) and for long (>8 h), versus referent sleep duration (mostly 7–8 h). The referent sleep duration group was similar to that used in other studies, except for Cao et al.[11], Shen et al.[13], and Wu et al.[4] where the baseline category was switched using the generalized least squares method [26]. In studies where multiple categories of short or long sleep were available [2, 5, 6, 8, 9, 13], a single estimate was obtained using the generalized least squares method [26]. We re-estimated RRs of breast cancer for <7, 7–8, >8 h of total sleep duration in the Million Women Study for the whole follow-up period and for the follow-up period with the first 5 years excluded, respectively. The meta-analysis calculated the inverse-variance weighted average of study-specific log RRs, which avoids giving disproportional weights to small-scale studies. Heterogeneity was assessed by Cochran’s Q test.

Subgroup analyses were performed for type of sleep duration (total sleep duration or nighttime sleep duration) and geographical region of the cohort (North America, Europe, and Asia), and heterogeneity was assessed by the *χ*^2^ test. As a sensitivity analysis, we restricted to 10 studies [2–4, 6–9, 12, 14] (including the Million Women Study) with analyses that excluded the early follow-up period or cases diagnosed soon after baseline. We performed a sensitivity analysis of the shortest versus average sleep of the individual studies. Since data on premenopausal women were only available in three studies [2, 3, 11], our sensitivity analysis was restricted to postmenopausal women [2–5, 11].

All statistical tests were two-sided. STATA 15.1 (StataCorp, College Station, TX) was used for all analyses and R (R Foundation for Statistical Computing, Vienna, Austria) for plotting forest plots.

## Results

### Million Women Study

Of the 713,150 women (mean age, 60 [*SD*, 5] years) included in these analyses, 23% reported ≤6 h of sleep, 68% reported 7–8 h of sleep, and 10% reported ≥9 h of sleep. A total of 36,173 breast cancer cases were diagnosed over a mean of 14.3 (*SD*, 3.8) years of follow-up, of which 24,476 cases were diagnosed after the first 5 years of follow-up.

In a subset of 13,109 women who returned a repeat questionnaire 1.7 (*SD*, 1.2) years after baseline, sleep duration (<7, 7–8, >8 h) showed moderate agreement (percentage of agreement: 76%; *κ* = 0.48). The Spearman non-parametric correlation was 0.66. When assessed as a continuous variable, the Bland–Altman plot showed good agreement of sleep duration in the two reports (mean difference: 0.01 h [95% limits of agreement: −2 to +2 h]).

Compared with women reporting sleeping 7–8 h, women reporting shorter sleep or longer sleep duration were more likely to be in the more deprived fifth of socioeconomic status, to have a higher BMI, to smoke, to rate their health as being poor, and to perform strenuous exercise less frequently, but were less likely to report high alcohol intake (*p* < 0.05 for all). The prevalence of daytime napping, current smoking, and not being in paid work were the highest among women sleeping >9 h. Most reproductive risk factors were similar across sleep duration categories (Table 1).

**Table 1.** Characteristics of 713,150 Million Women Study participants, by categories of self-reported sleep duration

	24-h sleep duration (h)				
	<6	6	7–8	9	>9
Characteristics, mean (*SD*) or %	*N* = 32,233 (4.5%)	*N* = 128,603 (18.0%)	*N* = 483,974 (67.9%)	*N* = 50,199 (7.0%)	*N* = 18,141 (2.5%)
Age at baseline (year)	59.7 (4.9)	59.6 (4.9)	59.6 (4.8)	60.5 (4.9)	60.5 (5.0)
Tertiary qualifications	12%	15%	17%	14%	12%
Socioeconomic status, the most deprived fifth	22%	18%	15%	16%	22%
Lifestyle and anthropometric factors					
Daytime napper	39%	40%	42%	56%	72%
BMI (kg/m^2^)	26.5 (4.9)	26.1 (4.6)	25.7 (4.3)	26.2 (4.4)	27.0 (5.1)
Height (cm)	161.7 (6.7)	162.2 (6.6)	162.5 (6.5)	162.3 (6.5)	161.9 (6.8)
Current smoker	12%	12%	12%	12%	15%
Alcohol intake, 7+ drinks per week	23%	27%	28%	27%	22%
Strenuous exercise per week, at least weekly	38%	42%	45%	40%	33%
Reproductive and hormonal factors					
Age at menarche (year)	12.9 (1.7)	12.9 (1.6)	13.0 (1.5)	13.1 (1.6)	13.1 (1.7)
Nulliparous	12%	11%	11%	11%	12%
Age at first birth (for parous women), year	23.5 (4.3)	24.0 (4.3)	24.3 (4.3)	23.9 (4.1)	23.5 (4.2)
Current HRT use	26%	27%	28%	29%	30%
Family history					
First-degree relative with breast cancer	11%	11%	11%	11%	11%
Health indicator					
Self-rated poor or fair health	38%	25%	16%	21%	38%

BMI, body mass index; HRT, hormone replacement therapy.

After exclusion of the first 5 years of follow-up, the multivariable-adjusted RRs of breast cancer for <6, 6, 9, and >9 h of sleep versus 7–8 h of sleep were 1.01 (0.95–1.07), 0.99 (0.96–1.03), 1.01 (0.96–1.06), and 1.03 (0.95–1.12), respectively (Supplementary Table S2). Breast cancer risk did not vary for each hour increase in sleep duration (RR = 1.01, 0.99–1.02). Sensitivity analyses yielded similarly null results, including those restricted to women who reported good/excellent health, and who reported never/rarely napping, and a complete case analysis (Supplementary Table S2). The RRs of breast cancer for <6, 6, 9, and >9 h of sleep versus 7–8 h of sleep when we restricted the valid sleep duration to 4–20 h were 1.00 (0.94–1.06), 0.99 (0.96–1.03), 1.01 (0.96–1.06), and 1.03 (0.95–1.12), respectively.

#### Meta-analysis

The search strategy identified a total of 743 records (Supplementary Figure S2). After excluding 240 duplicates, 503 titles and abstracts were screened. Of these, 19 full-text articles were assessed for eligibility. Six records were excluded, two of which lacked the exposure of interest [27, 28], one involved a patient population [29], one reported results that were updated in a subsequent publication [30], one did not provide sufficient information [31], and one had no full-text available [32]. One additional study was found online that was not identified through keyword searches in Embase and MEDLINE. We also included the Multiethnic cohort results, which were published subsequent to the literature search [14].

Our systematic review identified 14 previously published prospective studies (Supplementary Table S3) [1–14]. Together with the current Million Women Study results, these 15 studies included nearly 1.5 million women, with mean age ranging from 37 to 63 years. The average follow-up periods ranged from 7 to 18 years. The risk of biases in the included studies were low (Supplementary Table S4).

**Figure 1.**
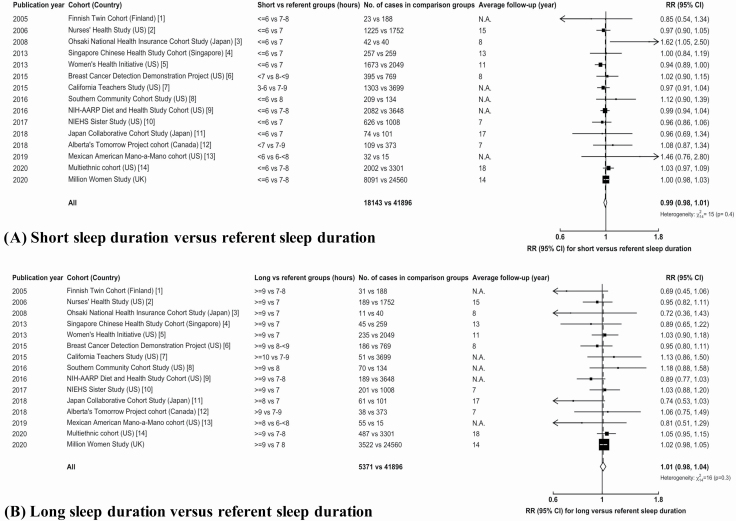
Meta-analysis of prospective studies on the risk of breast cancer in women for (A) short versus referent sleep duration and (B) long versus referent sleep duration. Study-specific RRs are represented by squares (with their 95% CIs as horizontal lines), with an area inversely proportional to the variance of study-specific log RR. The overall estimate is presented as a white diamond, obtained by the inverse-variance weighted averages of the log RRs of all analyzed studies. N.A., not available; RR, relative risk; CI, confidence interval.

When relevant results from the 15 studies were combined (based on data from 65,410 of these cases), the overall RRs for short (18,143 cases) or long (5,371 cases) sleep durations, respectively, compared to average sleep duration (41,896 cases) were 0.99 (0.98–1.01) and 1.01 (0.98–1.04) (Figure 1). Although the Million Women Study dominates these findings, the RRs excluding the Million Women Study results were 0.99 (0.96–1.01) and 0.99 (0.94–1.04) for short sleep and for long sleep versus average sleep duration, respectively, which were similar to the overall results. Combining available estimates from studies with exclusion of the first few years of follow-up or cases diagnosed soon after baseline yielded similar findings (Supplementary Figure S3).

Our subgroup analyses did not show statistically significant heterogeneity by geographical region (North America, Europe, and Asia; *p* for heterogeneity for short sleep vs average sleep = 0.6; *p* for heterogeneity for long sleep vs average sleep = 0.1) or type of sleep duration (nighttime sleep duration, total sleep duration over a 24-h period; *p* for heterogeneity for short sleep vs average sleep = 0.2; *p* for heterogeneity for long sleep vs average sleep = 0.5). The RR for long sleep versus average sleep duration among postmenopausal women in six studies was 1.02 (0.98–1.05), whereas that of short sleep duration was 0.99 (0.97–1.01). The RRs for shortest average sleep duration were 0.99 (0.96–1.02) for 15 studies and 1.00 (0.97–1.03) for 10 studies with analyses excluding the first few years of follow-up or cases diagnosed soon after baseline.

## Discussion

We did not find an association of hours of sleep, or of short or long sleep duration, with breast cancer risk in the Million Women Study or in a meta-analysis of 15 prospective studies, which included a total of 65,410 breast cancer cases. Our meta-analysis included data from 6 more prospective studies with a total of ~1 million more participants and ~45,000 more breast cancer cases than the most recent meta-analyses, published in 2018 [16]. The results of the meta-analysis were robust across different sensitivity analyses; for example, when restricted to findings that minimized reverse causation bias by excluding cases diagnosed soon after baseline. We also minimized potential biases associated with differential reporting of usual sleep duration by excluding studies with retrospective reporting of sleep duration.

The putative mechanisms linking sleep duration to breast cancer risk suggest different effects between short sleep duration and long duration sleep [17, 33] and we pre-specified our analyses to use categorical duration to examine the shape of any relationship. A study in the UK Biobank cohort was therefore not included in this meta-analysis, as results were not presented by categories of sleep duration. However, when self-reported sleep duration was modeled as a continuous variable in UK Biobank (with an average of 3 years of follow-up), results also suggested no association with overall breast cancer risk (RR for each hour of sleep = 1.00 [0.96–1.04]) [24]. The UK Biobank study also presented Mendelian randomization analyses with a genetic instrument for sleep duration and found inconsistent results [24]. Further studies using genetic instruments for sleep duration might help to clarify possible associations.

The strengths of the Million Women Study include the prospective design, adjustment for many potential confounding factors, the large number of cases, and a long period of follow-up. Sleep duration was assessed using repeated measurements. We found that a single question of usual sleep duration is a reasonably reliable measure of self-reported sleep duration, with some regression to the mean, as would be expected, and consistent with findings from other studies that individuals with shorter sleep tended to underestimate duration whereas those with longer sleep tended to overestimate duration [34–36]. Some reported that there was overall overestimation of sleep duration [37, 38], but this would not affect comparisons across categories of duration. The findings of the meta-analysis were consistent even when the Million Women Study results were not included.

This study also has some limitations. One of the main hypotheses proposed to explain an association between sleep duration and breast cancer risk is the light-at-night hypothesis, however, we studied total sleep duration in the Million Women Study as it was not possible to separate daytime napping duration from total sleep duration. Nonetheless, the results for total sleep duration were similarly null when we excluded women who reported napping. Furthermore, although short total sleep duration has been considered a surrogate for greater light-at-light exposure, many factors (such as the proportion of total sleep duration as daytime napping, awakening at night, indoor light exposure at night, and daytime sleep due to night shift work) may affect the likelihood of short sleep being associated with greater exposure to light at night. Overall though, evidence from meta-analyses of the associations of night shift work [39] and insomnia [40] with breast cancer risk, and from prospective studies of individual-level data on light-at-night exposures [10, 41, 42] does not suggest that these factors are related to breast cancer etiology. Further, little or no evidence for the carcinogenicity of long or short sleep duration is provided by evidence from meta-analyses of data on other sex hormone-related cancers (including endometrial cancer, ovarian cancer, and prostate cancer), although there are relatively few published data for endometrial cancer and for ovarian cancer [16].

In summary, despite the different populations studied and the inclusion and exclusion criteria for each prospective study in the meta-analyses, there is no heterogeneity in the findings across studies. The totality of the evidence thus suggests little or no effect of sleep duration on breast cancer risk.

## Supplementary Material

zsaa166_suppl_Supplemenarty_Materials
