# Supplementary material for: Sleep duration and breast cancer incidence: results from the Million Women Study and meta-analysis of published prospective studies
Source: Sleep. 2020 Sep 4;44(2):zsaa166. doi: 10.1093/sleep/zsaa166 (PMC7879408; doi:10.1093/sleep/zsaa166)
Supplement: zsaa166_suppl_Supplemenarty_Materials [file zsaa166_suppl_supplemenarty_materials.docx]

**Supplementary file**

**Sleep duration and breast cancer incidence: Results from the Million Women Study and meta-analysis of published prospective studies**

##### Angel T.Y. Wong^1^, Alicia K. Heath^2^, Tammy Y.N. Tong^1^, Gillian K. Reeves^1^, Sarah Floud^1^, Valerie Beral^1^, Ruth C. Travis^1^ on behalf of the Million Women Study

**List of tables and figures**

Table S1. Search terms of the meta-analysis of sleep duration with breast cancer incidence

Table S2. Relative risk and 95% confidence intervals for breast cancer by sleep duration in 713,150 Million Women Study participants: effect of individual and simultaneous adjustment

Table S3. Study characteristics of the 15 studies included in the meta-analysis

Table S4. Risk of bias assessment of the 15 studies included in the meta-analysis

Figure S1. Flow chart of exclusion of Million Women Study participants

Figure S2. Selection of studies for meta-analysis of sleep duration with breast cancer risk

Figure S3. Meta-analysis of prospective studies on the risk of breast cancer in women for (A) short versus referent sleep duration and (B) long versus referent sleep duration after exclusion of early follow-up periods or cases diagnosed soon after baseline.

Table S1. Search terms of the meta-analysis of sleep duration with breast cancer incidence

**MEDLINE**

1. Sleep/ or sleep*.mp.
2. sleep deprivation.mp. or Sleep Deprivation/
3. (nap or napping or daytime somnolence or siesta).mp. [mp=title, abstract, original title, name of substance word, subject heading word, keyword heading word, protocol supplementary concept word, rare disease supplementary concept word, unique identifier, synonyms]
4. breast cancer.mp. or Breast Neoplasms/
5. (breast carcinoma or breast neoplasm*).mp. [mp=title, abstract, original title, name of substance word, subject heading word, keyword heading word, protocol supplementary concept word, rare disease supplementary concept word, unique identifier, synonyms]
6. (prospective stud* or cohort stud* or longitudinal stud* or follow-up stud* or case-control stud*).mp. [mp=title, abstract, original title, name of substance word, subject heading word, keyword heading word, protocol supplementary concept word, rare disease supplementary concept word, unique identifier, synonyms]
7. 1 or 2 or 3
8. 4 or 5
9. 6 and 7 and 8
10. limit 9 to English language

**Embase**

1. sleep*.mp. or sleep/
2. sleep deprivation.mp. or sleep deprivation/
3. (nap or napping or daytime somnolence or siesta).mp. [mp=title, abstract, heading word, drug trade name, original title, device manufacturer, drug manufacturer, device trade name, keyword, floating subheading word]
4. breast neoplasm*.mp. or breast neoplasms/
5. breast cancer.mp. or breast cancer/
6. breast carcinoma.mp. or breast tumor/ or breast carcinoma/
7. (prospective stud* or cohort stud* or longitudinal stud* or follow-up stud* or case-control stud*).mp. [mp=title, abstract, heading word, drug trade name, original title, device manufacturer, drug manufacturer, device trade name, keyword, floating subheading word]
8. 1 or 2 or 3
9. 4 or 5 or 6
10. 7 and 8 and 9
11. limit 10 to English language

Figure S1. Flow chart of exclusion of Million Women Study participants in the current analysis


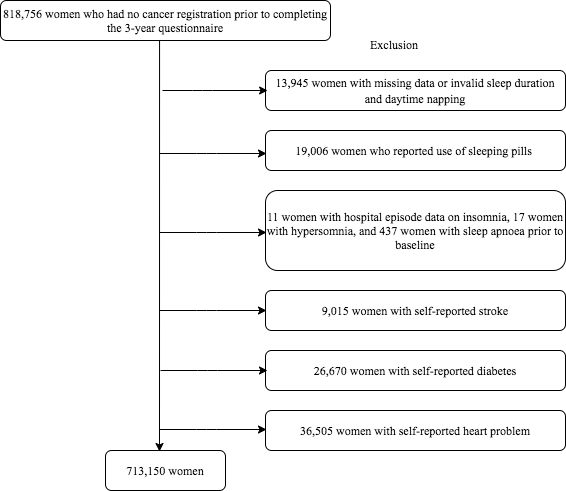


Figure S2. Selection of studies for meta-analysis of sleep duration with breast cancer risk


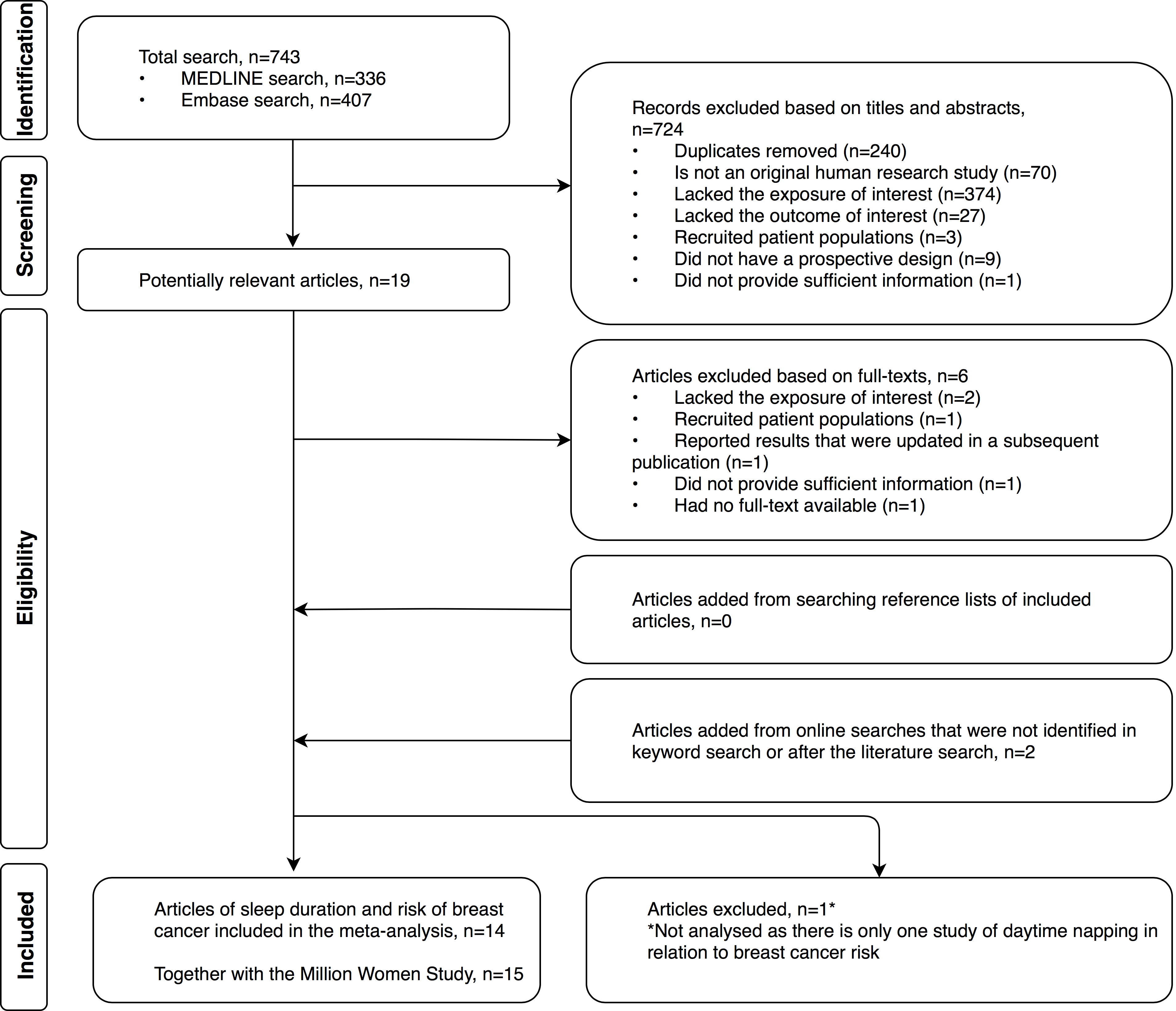


In total 15 studies, including the Million Women Study, were included. [1-14]

Table S2. Relative risk and 95% confidence intervals for breast cancer by sleep duration in 713,150 Million Women Study participants: effect of individual and simultaneous adjustment

|  |  | **RR (95% CI) of breast cancer for sleep duration categories (hours)** | | | | **P for heterogeneity** |
| --- | --- | --- | --- | --- | --- | --- |
|  | **<6** | **6** | **7-8** | **9** | **>9** |  |
| Minimally adjusted for region, stratified by year of birth and year of return of the questionnaire | 1.00 (0.94 - 1.06) | 0.99 (0.96 - 1.03) | 1.00 (-) | 1.02 (0.97 - 1.07) | 1.07 (0.98 - 1.15) | 0.5 |
| Additionally separately adjusted for: |  |  |  |  |  |  |
| Educational attainment | 1.01 (0.95 - 1.08) | 1.00 (0.96 - 1.03) | 1.00 (-) | 1.03 (0.98 - 1.08) | 1.08 (1.00 - 1.17) | 0.3 |
| Socioeconomic status | 1.01 (0.95 - 1.07) | 1.00 (0.96 - 1.03) | 1.00 (-) | 1.02 (0.97 - 1.07) | 1.07 (0.99 - 1.16) | 0.5 |
| Age at menarche | 1.00 (0.94 - 1.06) | 0.99 (0.96 - 1.03) | 1.00 (-) | 1.02 (0.97 - 1.07) | 1.07 (0.99 - 1.16) | 0.5 |
| Parity and age at first birth | 1.01 (0.95 - 1.07) | 1.00 (0.96 - 1.03) | 1.00 (-) | 1.02 (0.97 - 1.08) | 1.08 (0.99 - 1.16) | 0.4 |
| Body mass index | 0.99 (0.93 - 1.05) | 0.99 (0.95 - 1.02) | 1.00 (-) | 1.01 (0.96 - 1.06) | 1.04 (0.96 - 1.13) | 0.7 |
| Alcohol intake | 1.01 (0.95 - 1.07) | 1.00 (0.96 - 1.03) | 1.00 (-) | 1.02 (0.97 - 1.07) | 1.08 (1.00 - 1.17) | 0.4 |
| Smoking status and cigarettes per day | 1.00 (0.94 - 1.06) | 0.99 (0.96 - 1.02) | 1.00 (-) | 1.02 (0.97 - 1.07) | 1.06 (0.98 - 1.15) | 0.5 |
| Strenuous exercise per week | 1.00 (0.94 - 1.06) | 0.99 (0.96 - 1.03) | 1.00 (-) | 1.02 (0.97 - 1.07) | 1.06 (0.98 - 1.15) | 0.6 |
| Family history of breast cancer | 1.00 (0.94 - 1.06) | 0.99 (0.96 - 1.03) | 1.00 (-) | 1.02 (0.97 - 1.07) | 1.06 (0.98 - 1.15) | 0.5 |
| HRT use | 1.00 (0.94 - 1.06) | 0.99 (0.96 - 1.03) | 1.00 (-) | 1.01 (0.97 - 1.07) | 1.06 (0.97 - 1.14) | 0.7 |
| Height | 1.01 (0.95 - 1.07) | 1.00 (0.96 - 1.03) | 1.00 (-) | 1.02 (0.97 - 1.07) | 1.07 (0.99 - 1.16) | 0.5 |
| Napping frequency | 1.00 (0.94 - 1.07) | 1.00 (0.96 - 1.03) | 1.00 (-) | 1.01 (0.96 - 1.06) | 1.03 (0.95 - 1.12) | 0.9 |
|  |  |  |  |  |  |  |
| **All factors simultaneously** | **1.01 (0.95 - 1.07)** | **0.99 (0.96 - 1.03)** | 1.00 (-) | **1.01 (0.96 - 1.06)** | **1.03 (0.95 - 1.12)** | 0.9 |
|  |  |  |  |  |  |  |
| Sensitivity analyses for the multivariable-adjusted model: |  |  |  |  |  |  |
| Among women reporting good/excellent health | 1.04 (0.96 - 1.12) | 0.99 (0.95 - 1.03) | 1.00 (-) | 1.01 (0.95 - 1.07) | 1.05 (0.94 - 1.16) | 0.7 |
| Among women reporting never/rarely napping | 1.00 (0.92 - 1.08) | 0.96 (0.92 - 1.01) | 1.00 (-) | 0.98 (0.91 - 1.06) | 0.97 (0.83 - 1.13) | 0.6 |
| Complete case analysis | 1.02 (0.94 - 1.10) | 1.00 (0.96 - 1.04) | 1.00 (-) | 0.99 (0.93 - 1.05) | 1.09 (0.99 - 1.21) | 0.5 |

All analyses excluded the first five years of follow-up as pre-specified. BMI: body mass index. HRT: Hormone replacement therapy

| Table S3. Study characteristics of the 15 studies included in the meta-analysis | | | | | | | | |
| --- | --- | --- | --- | --- | --- | --- | --- | --- |
| **Cohort** (region, year of recruitment and baseline sleep duration, follow-up) | **Analysed population** (analysed sample size, baseline age, exclusion criteria) | | **Outcome** (ascertainment) | **Exposure**  (assessment) | **Exposure categories (hours)** | **RR** | **95% CI** | **Covariates** |
| **Night-time sleep duration** | | | | | | | | |
| **Alberta’s Tomorrow Project Cohort** (**Canada)** [12]  Recruited during 2001 - 2015  Sleep duration obtained in 2004 or 2008 - 2015  Follow-up: Baseline - June 2017 (mean, 7.2 y by calculation) | | 29,427 women aged 35-69 y at recruitment  Mean age at baseline: 53.0 y  Excluded women who were pregnant at the time of enrolment, did not consent to data linkage, had a personal history of cancer other than non-melanoma skin cancer, or had missing or invalid sleep duration | Breast cancer  Linkage to cancer registry | Survey 2004: “On average, how many hours did you sleep each night during the past 4 weeks?”  Survey 2008: “On average over the past 7 days, at what time did you normally go to sleep?” and “On average over the past 7 days, at what time did you normally wake up?”  The Updated Health and Lifestyle Questionnaire and the CORE Questionnaire from 2008 to 2015: “On average, how many hours per day do you usually sleep, including naps? A day refers to a 24-hour period.” ^Φ^  Questionnaires | Total (520 cases)  <7 (109)  7-9 (373)^Ω^  >9 (38) | 1.08  1.00  1.06 | 0.87-1.34  Ref  0.75-1.49 | Age, total household income, employment status, marital status, education, ethnicity, alcohol intake, smoking status, body mass index, presence of at least one medical conditions, presence of depression, family history of cancer, menopausal status, and gravidity |
|  |  |  |  |  | Excluding cases diagnosed in the first 2 y after enrolment (392 cases)  <7 (79)  7-9 (288)^Ω^  >9 (25) | 1.04  1.00  0.90 | 0.81-1.33  Ref  0.59-1.37 |  |
| **NIEHS Sister Study (US)** [10]  Recruited in 2003 - 2009  Sleep duration obtained at baseline  Follow-up: Baseline - 31^st^ August 2015 (mean, 7.4 y) | | 50,454* women aged 35-74 y at recruitment who had a sister diagnosed with breast cancer  Mean age at baseline: 55.6 y  Excluded women with a diagnosis of invasive or DCIS breast cancer prior to baseline, and those who were blind or were current shift workers | DCIS or invasive breast cancer  Annual health updates, follow-up questionnaires, 81% self-reported cases with medical records | Usual sleep duration  Updated information obtained during 2012-2014  Computer-assisted telephone interviews | Total (2,730^*^ cases)  ≤6 (626)  7 (1,008)  8 (895)  ≥9 (201) | 0.96  1.00  0.90  1.03 | 0.86-1.06  Ref  0.82-0.99  0.88-1.20 | Attained age as the underlying time variable, adjusted for race, education, household income, marital status, HRT use, use of oral contraceptives, alcohol consumption, age at menarche, parity, age at first birth, age at menopause, pack-years of smoking, and metabolic equivalent hours of physical activity per week |
|  |  |  |  |  | ER + breast cancer (1,569 cases)  ≤6 (321)  7 (593)  8 (534)  ≥9 (121) | 0.87  1.00  0.90  1.06 | 0.76-1.00  Ref  0.79-1.01  0.87-1.29 |  |
|  |  |  |  |  | ER - breast cancer (280 cases)  ≤6 (80)  7 (92)  8 (90)  ≥9 (18) | 1.26  1.00  1.05  0.94 | 0.92-1.73  Ref  0.78-1.42  0.54-1.64 |  |
| **NIH-AARP Diet and Health Study Cohort (US)** [9]  Recruited during 1995 - 1996  Sleep duration obtained 1 y after recruitment  Follow-up: Baseline - December 2006 | | 123,858 women aged 50-71 y at recruitment  Mean age at baseline: 62.6 y  Excluded women who died or moved out of study area before the risk factor questionnaire scan, were with missing sleep information, with prevalent cancers, or without diagnosis date, and completed questionnaire by proxy | Invasive breast cancer  Linkage to state cancer registry | “During a typical 24-hour period over the past 12 months, how many hours did you spend sleeping at night?”  Questionnaires | Total (5,919 cases)  <5 (162)  5-6 (1,920)  7-8 (3,648)  ≥9 (189) | 0.84  1.00  1.00  0.89 | 0.71-0.98  0.94-1.05  Ref  0.77-1.03 | Age, napping, race, education, marital status, self-reported health, family history of cancer, smoking, physical activity, sitting time, diabetes, hypertension, BMI, NSAID use, alcohol drinking, intakes of fruits and vegetables, wholegrain, total fat, red meat and total calories, postmenopausal hormonal use, menopausal status, number of live child birth, oral contraception use, hysterectomy, and oophorectomy |
|  |  |  |  |  | Excluding cases diagnosed in the first 2 y after enrolment (4,660 cases)  <5 (124)  5-6 (1,526)  7-8 (2,864)  ≥9 (146) | 0.80  1.00  1.00  0.89 | 0.67-0.96  0.94-1.06  Ref  0.75-1.05 |  |
| **Women’s Health Initiative (US)** [5]  Recruited in 1993 - 1998^§^  Sleep duration obtained at baseline  Follow-up: Baseline - 30^th^ September 2010 (mean, 10.8 y by calculation) | | 110,011 postmenopausal women aged 50-79 y at recruitment  Mean age at baseline: 62.9 y  Excluded women with a prior cancer (except non-melanoma skin cancer), missing information on prior cancer, no information on last follow-up or last contact, no information on the primary sleep exposures, or missing data on at least one adjusted covariate | Invasive breast cancer  Medical record and pathology report review with final verification at the Clinical Coordinating Centre  Deaths confirmed by death certificates | “About how many hours of sleep did you get in a typical night during the past 4 weeks?”  Interviews at screening visits | Total (5,149 cases)  ≤5 (355)  6 (1,318)  7 (2,049)  8 (1,192)  ≥9 (235) | 0.95  0.94  1.00  0.99  1.03 | 0.85-1.07  0.87-1.00  Ref  0.92-1.06  0.90-1.18 | Age, clinical trial arm assignment, number of live births, age at menarche, age at menopause, BMI, energy expenditure, education, income, race/ethnicity, marital status, age at first birth, pervious use of HRT, history of benign breast disease, family history of breast cancer, alcohol consumption, and smoking status |
|  |  |  |  |  | ER+ breast cancer (4,013 cases)  ≤5 (256)  6 (1,013)  7 (1,598)  8 (959)  ≥9 (187) | 0.92  0.94  1.00  1.02  1.06 | 0.80-1.05  0.87-1.01  Ref  0.94-1.10  0.91-1.23 |  |
|  |  |  |  |  |  |  |  |  |
|  |  |  |  |  | ER- breast cancer (727 cases)  ≤5 (57)  6 (201)  7 (290)  8 (152)  ≥9 (27) | 0.95  0.96  1.00  0.91  0.86 | 0.71-1.28  0.80-1.15  Ref  0.75-1.11  0.58-1.28 |  |
| **Total 24-hour sleep duration** | | | | | | | | |
| **Breast Cancer Detection Demonstration Project (US)** [6]  Recruited in 1979  Sleep duration obtained in 1987 - 1989  Follow-up: Baseline - 31^st^ December 2005 (mean, 8.2 y by calculation) | | 40,013 women  Mean age at baseline: 61.8 y  Excluded women who reported existing cancer and those who did not report sleep duration | Breast cancer  Pathology reports, self-reports (mostly verified by pathology reports), and linkage to state cancer registries and the National Death Index | “Thinking about a typical weekday and weekend day during the past year, how many hours per day did you spend on sleeping?”^¶^  Questionnaires | Weekday (1,846 cases)  <6 (69)  6-<7 (326)  7-<8 (496)  8-<9 (769)  ≥9 (186) | 0.90  1.04  0.91  1.00  0.95 | 0.70-1.16  0.91-1.18  0.81-1.02  Ref  0.80-1.11 | Age, race, education, marital status, BMI, vigorous physical activity, smoking status, pack-year, year since quitting, number of live birth, age at first live birth, age at menarche, menopausal status, use of HRT, use of multivitamin, history of diabetes, family history of cancer, and alcohol consumption |
|  |  |  |  |  | Weekend (1,713 cases)  <6 (44)  6-<7 (176)  7-<8 (357)  8-<9 (809)  ≥9 (327) | 0.86  0.92  0.91  1.00  0.99 | 0.63-1.17  0.78-1.08  0.80-1.03  Ref  0.87-1.12 |  |
|  |  |  |  |  | Weighted average^¶^ (1,553 cases)  <6 (45)  6-<7 (267)  7-<8 (449)  8-<9 (631)  ≥9 (161) | 0.87  1.04  0.93  1.00  1.00 | 0.64-1.18  0.90-1.20  0.82-1.05  Ref  0.84-1.19 |  |
|  |  |  |  |  | Weekday, ER + breast cancer (839 cases)  <6 (18)  6-<7 (124)  7-<8 (221)  8-<9 (378)  ≥9 (98) | 0.53  0.83  0.82  1.00  1.04 | 0.33-0.86  0.68-1.02  0.69-0.97  Ref  0.83-1.30 |  |
|  |  |  |  |  | Weekday, ER - breast cancer (188 cases)  <6 (14)  6-<7 (25)  7-<8 (55)  8-<9 (73)  ≥9 (21) | 2.12  0.84  1.02  1.00  1.17 | 1.18-3.82  0.53-1.33  0.72-1.45  Ref  0.72-1.91 |  |
|  |  |  |  |  | Weekday, excluding cases diagnosed within the first 2y of follow-up (1,489 cases)  <6 (57)  6-<7 (257)  7-<8 (402)  8-<9 (624)  ≥9 (149) | 0.93  1.01  0.90  1.00  0.95 | 0.70-1.22  0.87-1.17  0.80-1.03  Ref  0.79-1.13 |  |
| **California Teachers Study (US)** [7]  Recruited in 1995 - 1996  Sleep duration information obtained at baseline  Follow-up: Baseline - 31^st^ December 2011 | | 101,609 female professional school employees aged 22-104 y at baseline  Mean age at baseline: 52 y  Excluded women who lived outside California at baseline, agreed to breast cancer research only, had an unknown history of prior cancer, had a prior history of invasive cancer, asked to be removed from study after joining or had unknown or invalid information about sleep duration (<3 hr/day) | Invasive breast cancer  Linkage to cancer registry | “In the past year, on average, how many hours per day did you spend sleeping”  Questionnaires | Total (5,053 cases)  3-6 (1,303)  7-9 (3,699)  ≥10 (51) | 0.97  1.00  1.13 | 0.91-1.04  Ref  0.86-1.50 | Ages at the start and end of follow-up (in days) as time on study, stratified by age, and adjusted for race/ethnicity, alcohol consumption, and menopausal status/hormone therapy use |
|  |  |  |  |  | Excluding case diagnosed in the first 2y after baseline (4,381 cases)  3-6 (1,139)  7-9 (3,196)  ≥10 (46) | 0.98  1.00  1.25 | 0.92-1.05  Ref  0.93-1.68 |  |
| **Finnish Twin Cohort (Finland)** [1]  Recruited in 1974  Sleep duration obtained in 1975  Follow-up: 1^st^ January 1976 - 31^st^ December 1996 | | 12,222 female twins born before 1958  Mean age at baseline: 36.5 y  Excluded women with missing data on sleep duration in both 1975 and 1981 questionnaires | Invasive or *in situ* breast cancer  Linkage to cancer registry | “How many hours do you usually sleep per 24 hours?”  Updated information obtained in 1981  Questionnaires | Total (242 cases)  ≤ 6 (23)  7-8 (188)  ≥9 (31) | 0.85  1.00  0.69 | 0.54-1.34  Ref  0.45-1.06 | Age, zygosity, social class, number of children, use of oral contraceptives, BMI, alcohol use, smoking status, and physical activity |
|  |  |  |  |  | Participants with the same sleep duration in 1975 and 1981(146 cases)  ≤6 (14)  7-8 (125)  ≥9 (7) | 1.10  1.00  0.28 | 0.59-2.05  Ref  0.09-0.88 |  |
| **Japan Collaborative Cohort Study** **(Japan)** [11]  Recruited during 1988 - 1990  Sleep duration obtained at baseline  Follow-up: Baseline - 2009 (mean, 16.9 y by calculation) | | 34,350 women aged 40-79 y at recruitment  Mean age at baseline: 61.1 y  Excluded women with previous diagnosis of breast cancer or missing data on sleep duration | Breast cancer  Reviewed the records of local and major hospitals or collected from cancer registries | The average sleep duration on weekdays during the preceding year  Questionnaires | Total (236 cases)  ≤6 (74)  7 (101)  ≥8 (61) | 1.31  1.36  1.00 | 0.92-1.86  0.98-1.90  Ref | Adjusted for age, age at menarche, age at first child birth, BMI, parity, family history of breast cancer, marital status, sport time, walking time, alcohol intake, smoking status, hormone use, history of diabetes, and age and type of menopause |
|  |  |  |  |  | Premenopausal (163 cases)  ≤6 (46)  7 (74)  ≥8 (43) | 1.11  1.34  1.00 | 0.72-1.71  0.90-1.98  Ref |  |
|  |  |  |  |  | Postmenopausal (73 cases)  ≤6 (28)  7 (27)  ≥8 (18) | 1.98  1.49  1.00 | 1.08-3.70  0.81-2.76  Ref |  |
| **Mexican American Mano-a-Mano cohort** **(US)** [13]  Recruited since 2001  Sleep duration obtained in 2001 - 2012 ^Ω^  Follow-up: 10.6 y (median) ^Ω^ | | 8,637 women aged 20-60 y at recruitment  Mean age at baseline: 41 y ^Ω^  Women did not have self-reported diagnosis of cancer, diabetes, or cardiovascular diseases at the time of enrolment | Breast cancer  Updated by annual telephone call and confirmed with Taxes Cancer Registry | 24-hour sleep duration  Face-to-face interviews | Total (102 cases)  <6 (32)  6-<8 (15)  8-<9 (42)  ≥9 (13) | 1.71  1.17  1.00  1.38 | 0.92-3.18  0.73-1.87  Ref  0.69-2.74 | Adjusted for birthplace, language acculturation, age, marital status, education level, smoking status, drinking status, sitting time, physical activity, and BMI category |
| **Million Women Study (UK)**  Recruited in 1996 - 2001  Sleep duration obtained in 1999 - 2005  Follow-up: Baseline - 31^st^ December 2016 or 2017 (mean, 14.3 y) | | 713,150 women aged 50-64 y at recruitment  Mean age at baseline: 60.0y  (mostly postmenopausal at baseline)  Excluded women with a diagnosis of cancer, heart disease, stroke, diabetes, or sleep disorders prior to baseline, or incomplete or invalid data on sleep patterns, or reported use of sleeping pills | Invasive breast cancer  Linkage to cancer registries | “About how many hours sleep do you get in every 24 hours? (Please include naps)”  Questionnaires | Total (36,173 cases)  ≤6 (8,091)  7-8 (24,560)  >8 (3,522) | 1.00  1.00  1.02 | 0.98-1.03  Ref  0.98-1.05 | Stratified by year of baseline and year of birth, and adjusted for region at recruitment, educational attainment, Townsend deprivation index, BMI, alcohol intake, ever use of HRT and duration among current users, strenuous exercise at recruitment, smoking status and number of cigarettes, age at menarche, parity and age at first birth, family history of breast cancer, frequency of daytime napping, and height |
|  |  |  |  |  | Excluding the first 5 years of follow-up (24,476 cases)  ≤6 (5,459)  7-8 (16,672)  ≥9 (2,345) | 1.00  1.00  1.01 | 0.97-1.03  Ref  0.97-1.06 |  |
| **Multiethnic cohort (US) [14]**  Recruited in 1993 - 1996  Sleep duration obtained at baseline  Follow-up:  Baseline - 31^st^ December 2013  (mean, 17.5 y) | | 74,481 women aged 45-75 at recruitment  Age at baseline: 45-75 y  Excluded women who did not belong to the specified ethnic groups, had a previous breast cancer diagnosis, or had missing data on essential covariates | Invasive and *in situ* breast cancer  Linkage to cancer registry | “On the average, during the last year, how many hours in a day did you sleep (include naps)?”  Questionnaires | Total (5,790 cases)  ≤6 (2,002)  7-8 (3,301)  ≥9 (387) | 1.03  1.00  1.05 | 0.97-1.09  Ref  0.95-1.15 | Body mass index, ethnicity, age, education, family history of breast cancer, smoking status, alcohol use, physical activity, age at menarche, age at first live birth, number of children, hormone treatment, menopausal status, caffeine intake and total energy intake |
|  |  |  |  |  | Excluding cases diagnosed in the first 2 y after baseline (cases: NK)  ≤6  7-8  ≥9 | 1.03  1.00  1.04 | 0.97-1.09  Ref  0.94-1.15 |  |
| **Nurses’ Health Study (US)** [2]  Recruited in 1976  Sleep duration obtained in 1986  Follow-up: Baseline - 31^st^ May 2002 (mean, 14.9 y by calculation) | | 77,418 female registered nurses aged 30-55 y at recruitment  Mean age at baseline: 52.9 y  Excluded women with a diagnosis of cancer (except non-melanoma skin cancer) before baseline and those who did not provide information on sleep duration | Invasive breast cancer  Self-report and confirmed by blinded medical chart review or by the participants  Most breast cancer deaths identified by the next of kin or through National Death Index searches | Total hours of sleep in a 24-hour period  Updated information obtained in 2000  Questionnaires | Total (4,223 cases)  ≤5 (174)  6 (1,051)  7 (1,752)  8 (1,057)  ≥9 (189) | 0.93  0.98  1.00  1.05  0.95 | 0.79-1.09  0.91-1.06  Ref  0.97-1.13  0.82-1.11 | Age, BMI, height, history of benign breast disease, family history of breast cancer, parity and age at first birth, age at menarche, age at menopause, postmenopausal hormone use, physical activity, alcohol and caloric intake, and smoking |
|  |  |  |  |  | Premenopausal women (431 cases)  ≤5 (11)  6 (106)  7 (192)  8 (102)  ≥9 (20) | 0.74  1.05  1.00  0.93  0.92 | 0.39-1.41  0.82-1.33  Ref  0.73-1.19  0.57-1.47 |  |
|  |  |  |  |  | Postmenopausal women (3,620 cases)  ≤5 (157)  6 (893)  7 (1,487)  8 (921)  ≥9 (162) | 0.97  0.97  1.00  1.07  0.96 | 0.82-1.14  0.89-1.06  Ref  0.99-1.16  0.81-1.13 |  |
|  |  |  |  |  | Among participants who consistently reported the same number of hours of sleep in 1986 and 2000 (1,333 cases)  ≤5 (41)  6 (295)  7 (581)  8 (373)  ≥9 (43) | 1.06  1.04  1.00  1.22  1.17 | 0.77-1.46  0.90-1.20  Ref  1.07-1.39  0.85-1.60 |  |
|  |  |  |  |  | Excluding cases diagnosed <4 y after baseline (cases: *not given*)  ≤5 (N.A.)  6 ( N.A.)  7 ( N.A.)  8 ( N.A.)  ≥9 ( N.A.) | 0.94  0.94  1.00  1.04  0.90 | 0.79-1.12  0.86-1.03  Ref  0.95-1.14  0.76-1.08 |  |
| **Ohsaki National Health Insurance Cohort Study (Japan)** [3]  Recruited in 1994  Sleep duration obtained at baseline  Follow-up: 1995 - 2003 (mean, 7.7 y by calculation) | | 23,995 women aged 40-79 y at recruitment  Mean age at baseline: 60.8 y  Excluded women who had withdrawn from the National Health Insurance before follow-up, had history of cancer, had omitted responses for sleep duration, or had reported sleep duration <4 or >12 h | Breast cancer  Linkage to cancer registry | Mean integer number of hours of sleep per day during the last year  Questionnaires | Total (143 cases)  ≤6 (42)  7 (40)  8 (50)  ≥9 (11) | 1.62  1.00  1.14  0.72 | 1.05-2.50  Ref  0.75-1.73  0.36-1.43 | Age, BMI, history of diseases (stroke, hypertension, myocardial infarction or diabetes mellitus), family history of cancer, job, marital status, education, cigarette smoking, alcohol consumption, time spent walking, total caloric intake, menopausal status, age at menarche, age at first delivery, number of deliveries, use of oral contraceptive drugs, and use of hormone drugs except for oral contraceptive drugs |
|  |  |  |  |  | Premenopausal women (28 cases)  ≤6 (11)  7 (8)  8 (9)  ≥9 (0) | 2.06  1.00  1.48  *NA* | 0.81-5.23  Ref  0.56-3.93  *NA* |  |
|  |  |  |  |  | Postmenopausal women (101 cases)  ≤6 (28)  7 (29)  8 (35)  ≥9 (9) | 1.46  1.00  1.04  0.74 | 0.86-2.46  Ref  0.63-1.70  0.35-1.59 |  |
|  |  |  |  |  | Excluding cases diagnosed in the first 3 y of follow-up (94 cases)  ≤6 ( N.A.)  7 ( N.A.)  8 ( N.A.)  ≥9 ( N.A.) | 1.67  1.00  0.99  0.29 | 1.00-2.78  Ref  0.59-1.65  0.09-0.98 |  |
| **Singapore Chinese Health Study Cohort (Singapore)** [4]  Recruited in 1993 - 1998  Sleep duration obtained at baseline  Follow-up: Baseline - 31^st^ December 2008 (mean, 13.4 y by calculation) | | 34,028 women aged 45-74 y at recruitment  Mean age at baseline: 56 y estimated from a previous study [15]  Excluded women with prevalent cancer at baseline or incident breast cancer <2 y of follow-up | Breast cancer  Linkage to cancer registry | Average number of hours of sleep in a 24-hour period  Interview | Total (769 cases)  ≤6 (257)  7 (259)  8 (208)  ≥9 (45) | 1.00  1.00  1.00  0.89 | Ref  0.84-1.19  0.84-1.21  0.64-1.22 | Age at recruitment, year of recruitment, dialect group, education, age when period became regular, parity, BMI, and menopausal status |
|  |  |  |  |  | Postmenopausal women (515 cases)  ≤6 (193)  7 (162)  8 (129)  ≥9 (31) | 1.00  0.91  0.90  0.81 | Ref  0.74-1.12  0.72-1.13  0.55-1.18 |  |
| **Southern Community Cohort Study (US)** [8]  Recruited in 2002 - 2009  Sleep duration obtained at baseline  *Varying censoring dates among states*  *Nested case-control study* | | 518 cases and 42,435 controls nested in a cohort of 42,953 women aged 40-79 y at recruitment  Mean age at baseline: 52.2 y  Excluded women who did not have information on sleep duration, reported a previous diagnosis of cancer (except non-melanoma skin cancer) or resided outside the 11 states where cancer registry data were available | Invasive breast cancer  Linkage to state cancer registries | How many hours they typically slept in a 24-hour period, on weekdays and weekends separately  Questionnaires | Weekday sleep duration (515 cases)  <6 (86)  6 (123)  7 (102)  8 (134)  ≥9 (70) | 1.16  1.09  1.25  1.00  1.18 | 0.88-1.52  0.85-1.40  0.96-1.63  Ref  0.88-1.58 | Age, enrolment year, race, enrolment state, education, marital status, income, BMI, moderate-to-vigorous physical activity, overall sitting, smoking status, pack-year of smoking, number of live birth, age at first birth, length of breast feeding, age at menarche, menopause, use of menopausal hormone therapy, use of multivitamin, current use of aspirin, history of diabetes, family history of cancer, alcohol consumption, and dietary intakes of total fat, fibre, folate, and total calories |
|  |  |  |  |  | Weekend sleep duration (518 cases)  <6 (69)  6 (107)  7 (83)  8 (146)  ≥9 (113) | 1.01  1.11  1.09  1.00  1.11 | 0.75-1.35  0.86-1.43  0.83-1.44  Ref  0.87-1.43 |  |
|  |  |  |  |  | Weighted average^¶^ (**--** cases)^†^  <6 (89)  6 (118)  7 (113)  8 (127)  ≥9 (72) | 1.09  1.01  1.19  1.00  1.08 | 0.83-1.44  0.78-1.30  0.92-1.54  Ref  0.80-1.45 |  |
|  |  |  |  |  | Weekday, excluding cases diagnosed <2 y after baseline (379 cases)  <6 (64)  6 (95)  7 (75)  8 (96)  ≥9 (49) | 1.20  1.18  1.30  1.00  1.14 | 0.87-1.66  0.89-1.58  0.96-1.76  Ref  0.80-1.61 |  |
|  |  |  |  |  | Weekday, ER + breast cancer (201 cases)  <6 (33)  6 (47)  7 (34)  8 (56)  ≥9 (31) | 1.03  0.98  1.05  1.00  1.18 | 0.66-1.59  0.66-1.45  0.68-1.62  Ref  0.76-1.85 |  |
|  |  |  |  |  | Weekday, ER - breast cancer (110 cases)  <6 (24)  6 (27)  7 (24)  8 (20)  ≥9 (15) | 2.10  1.65  2.30  1.00  1.64 | 1.15-3.83  0.92-2.96  1.26-4.19  Ref  0.84-3.23 |  |

DCIS: ductal carcinoma *in situ*. ER +: oestrogen receptor positive. ER -: oestrogen receptor negative. NA: not applicable. NIH-AARP: National Institutes of Health-American Association of Retired Persons. NSAID: Nonsteroidal anti-inflammatory drugs. NIEHS: National Institute of Environmental Health Sciences. N.A.: Not available. RR: relative risk. CI: confidence interval.

^Ω^ Details were provided by the corresponding author via email.

^Φ^ We classified the type of exposure of the study as night-time sleep as two out of three sleep measures were night-time sleep and only one of them was total sleep duration.

^¶^ Weighted average was estimated by [(weekday sleep duration x 5) + (weekend sleep duration x 2)/7]

^*^ Calculated from the baseline descriptive table by usual sleep duration (in the article, the reported number of participants was 50,533 and the number of cases was 2,736.)

^†^ 519 cases in the article

^§^ Information was extracted from Prentice and Anderson [16]

Table S4. Risk of bias assessment of the 15 studies included in the meta-analysis

| **Study [reference]** | **Exposure** | | |  | **Comparability** | **Outcome** | | | **Total Max. 8** |
| --- | --- | --- | --- | --- | --- | --- | --- | --- | --- |
|  | Selection of non-exposed group | Absence of outcome at start of study | Exposure Ascertainment  (1 star for objective measures) | | Comparability  (1 star for adjusting for BMI and exogenous hormone use) | Outcome assessment  (1 star for record linkage/ adjudication) | Adequacy of follow-up  (1 star for record linkage to registry data) | Adequate follow-up period  (10 years or more) |  |
| Alberta’s Tomorrow Project cohort[12] | ★ | ★ |  | | ★ | ★ | ★ | ★ | 6 |
| Breast Cancer Detection Demonstration Project[6] | ★ | ★ |  | | ★★ | ★ | ★ | ★ | 7 |
| California Teachers Study[7] | ★ | ★ |  | |  | ★ | ★ | ★ | 5 |
| Finnish Twin Cohort[1] | ★ | NK |  | | ★ | ★ | ★ | ★ | 5 |
| Japan Collaborative Cohort study[11] | ★ | ★ |  | | ★★ | ★ | ★ | ★ | 7 |
| Mexican American Mano-a-Mano Cohort[13] | ★ | ★ |  | |  | ★ | ★ | ★ | 5 |
| Million Women Study (this study) | ★ | ★ |  | | ★★ | ★ | ★ | ★ | 7 |
| Multiethnic cohort[14] | ★ | ★ |  | | ★★ | ★ | ★ | ★ | 7 |
| NIEHS Sister Study[10] | ★ | ★ |  | | ★★ | ★ | ★ | ★ | 7 |
| NIH-AARP Diet and Health Study Cohort[9] | ★ | ★ |  | | ★★ | ★ | ★ | ★ | 7 |
| Nurses’ Health Study[2] | ★ | ★ |  | | ★★ |  | ★ | ★ | 6 |
| Ohsaki National Health Insurance Cohort Study[3] | ★ | ★ |  | | ★★ | ★ | ★ |  | 6 |
| Singapore Chinese Health Study Cohort[4] | ★ | ★ |  | | ★ | ★ | ★ | ★ | 6 |
| Southern Community Cohort Study[8] | ★ | ★ |  | | ★★ | ★ | ★ | NK | 6 |
| Women’s Health Initiative[5] | ★ | ★ |  | | ★★ | ★ | ★ | ★ | 7 |

NK: Not known due to insufficient information.

For comparability, an extra star was awarded for studies which adjusted for >3 of the following variables: alcohol intake, physical activity, age at menarche, menopausal status/age at menopause, parity, family history of breast cancer, and breastfeeding.

BMI: body mass index.

**
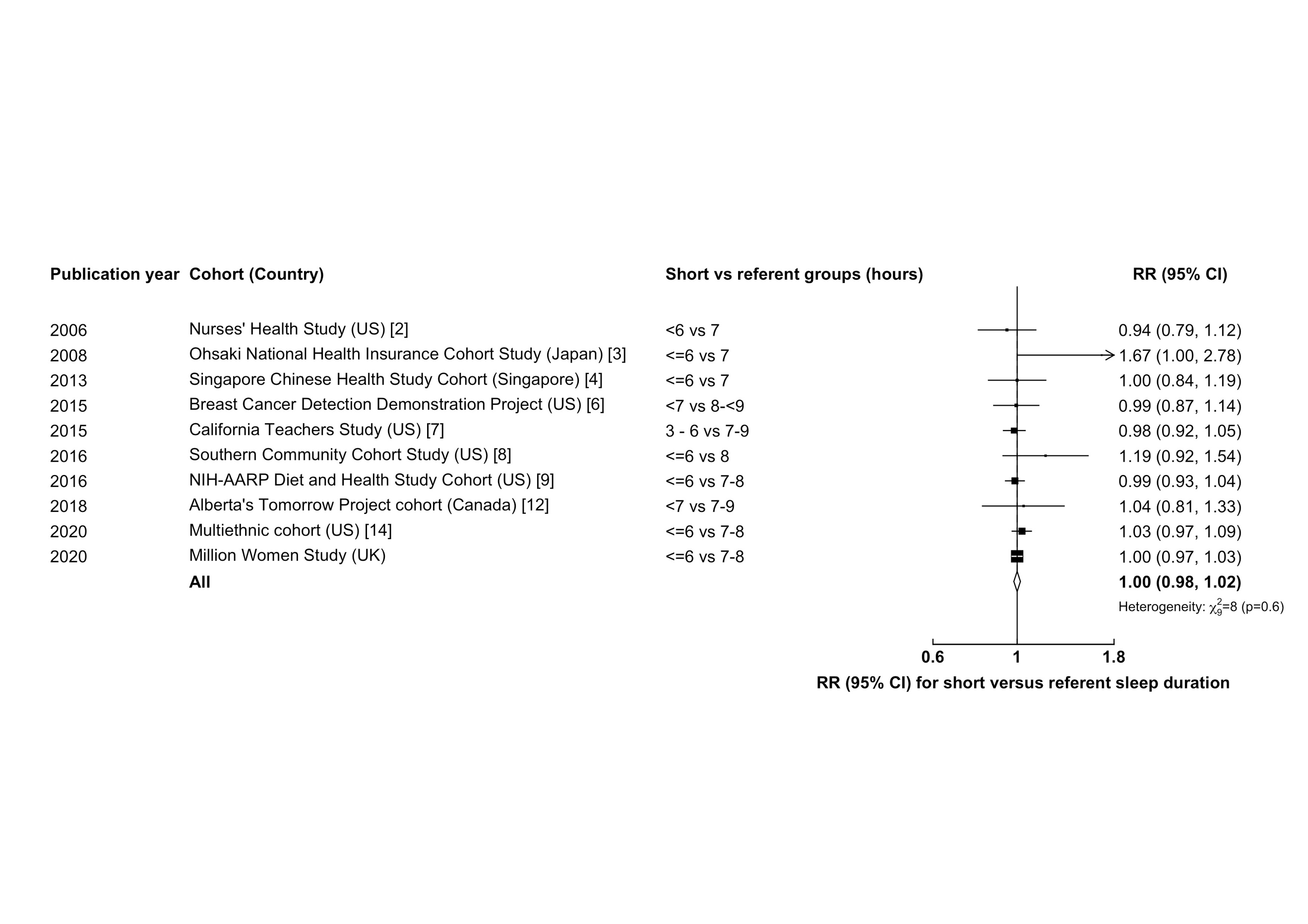
**

**(3A)** **Short sleep duration versus referent sleep duration**


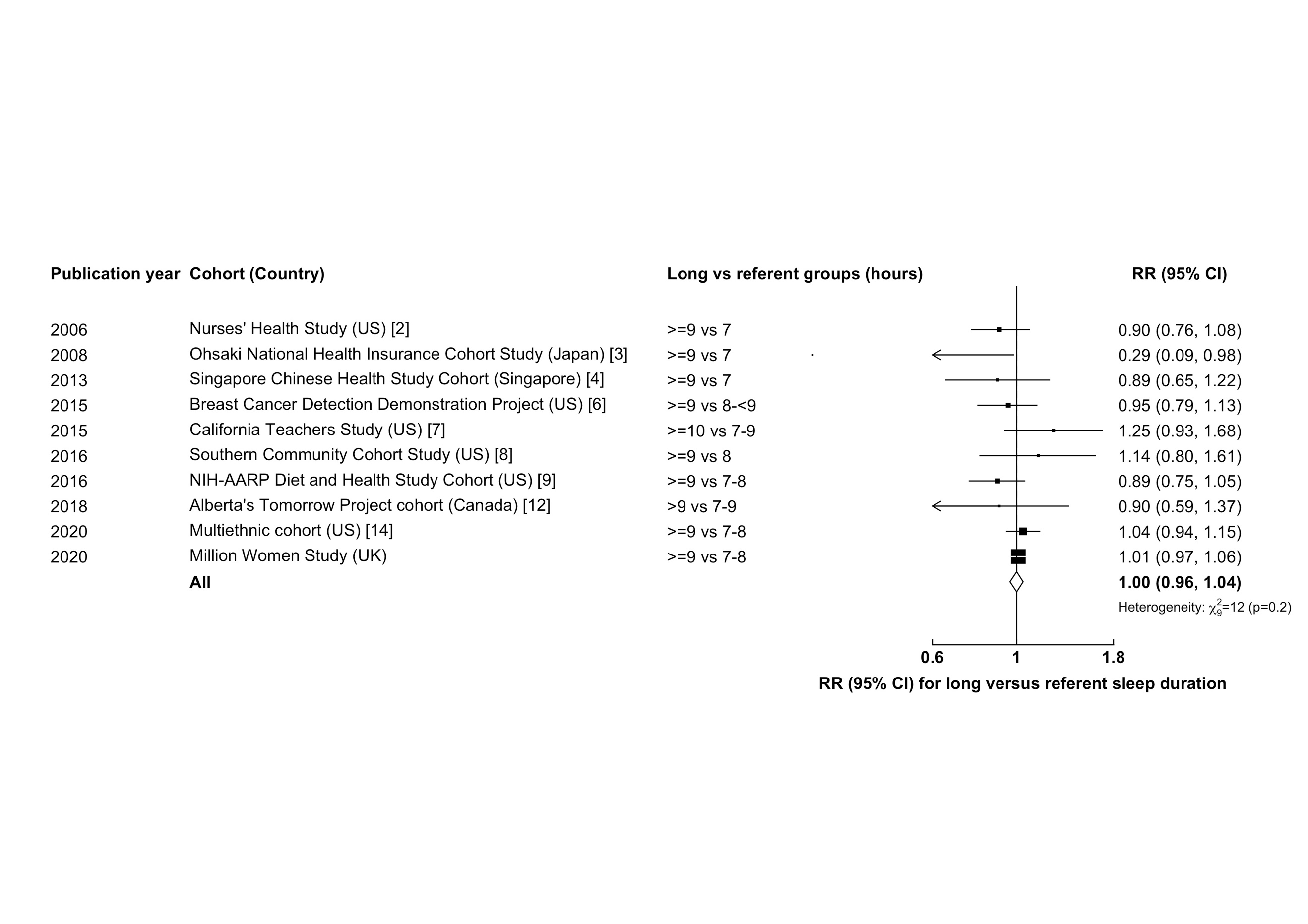


**(3B)** **Long sleep duration versus referent sleep duration**

Figure S3. Meta-analysis of prospective studies on the risk of breast cancer in women for (A) short versus referent sleep duration and (B) long versus referent sleep duration after exclusion of early follow-up periods or cases diagnosed soon after baseline

Study-specific RRs are represented by squares (with their 95% confidence intervals as horizontal lines); with an area inversely proportional to the variance of study-specific log RR. The overall estimate is presented as a white diamond, obtained by the inverse-variance weighted averages of the log RRs of all analysed studies. In the Nurses’ Health Study, as the number of cases in the baseline reference category was not provided, we included their results for the shortest sleep duration versus average sleep duration for the sensitivity analysis of short sleep duration.[2] RR: relative risk. CI: confidence interval.

References

1. Verkasalo PK, Lillberg K, Stevens RG, et al. Sleep duration and breast cancer: a prospective cohort study. Cancer Res. 2005;65(20):9595-600.

2. Pinheiro SP, Schernhammer ES, Tworoger SS, et al. A prospective study on habitual duration of sleep and incidence of breast cancer in a large cohort of women. Cancer Res. 2006;66(10):5521-5.

3. Kakizaki M, Kuriyama S, Sone T, et al. Sleep duration and the risk of breast cancer: the Ohsaki Cohort Study. Br J Cancer. 2008;99(9):1502-5.

4. Wu AH, Stanczyk FZ, Wang R, et al. Sleep duration, spot urinary 6-sulfatoxymelatonin levels and risk of breast cancer among Chinese women in Singapore. Int J Cancer. 2013;132(4):891-6.

5. Vogtmann E, Levitan EB, Hale L, et al. Association between sleep and breast cancer incidence among postmenopausal women in the Women's Health Initiative. Sleep. 2013;36(10):1437-44.

6. Qian X, Brinton LA, Schairer C, et al. Sleep duration and breast cancer risk in the Breast Cancer Detection Demonstration Project follow-up cohort. Br J Cancer. 2015;112(3):567-71.

7. Hurley S, Goldberg D, Bernstein L, et al. Sleep duration and cancer risk in women. Cancer Causes Control. 2015;26(7):1037-45.

8. Xiao Q, Signorello LB, Brinton LA, et al. Sleep duration and breast cancer risk among black and white women. Sleep Med. 2016;20:25-9.

9. Gu F, Xiao Q, Chu LW, et al. Sleep Duration and Cancer in the NIH-AARP Diet and Health Study Cohort. PLoS One. 2016;11(9):e0161561.

10. White AJ, Weinberg CR, Park YM, et al. Sleep characteristics, light at night and breast cancer risk in a prospective cohort. Int J Cancer. 2017;141(11):2204-14.

11. Cao J, Eshak ES, Liu K, et al. Sleep duration and risk of breast cancer: The JACC Study. Breast Cancer Res Treat. 2018;20:20.

12. McNeil J, Barberio AM, Friedenreich CM, et al. Sleep and cancer incidence in Alberta's Tomorrow Project cohort. Sleep. 2019;42(3).

13. Shen J, Chrisman M, Wu X, et al. Sleep duration and risk of cancer in the Mexican American Mano-a-Mano Cohort. Sleep Health. 2019;5(1):78-83.

14. Shigesato M, Kawai Y, Guillermo C, et al. Association between sleep duration and breast cancer incidence: The multiethnic cohort. Int J Cancer. 2020;146(3):664-70.

15. Wu AH, Wang R, Koh WP, et al. Sleep duration, melatonin and breast cancer among Chinese women in Singapore. Carcinogenesis. 2008;29(6):1244-8.

16. Prentice RL, Anderson GL. The women's health initiative: lessons learned. Annu Rev Public Health. 2008;29:131-50.
